# ^1^H-NMR Metabolomics Identifies Significant Changes in Metabolism over Time in a Porcine Model of Severe Burn and Smoke Inhalation

**DOI:** 10.3390/metabo9070142

**Published:** 2019-07-12

**Authors:** Cole Hendrickson, Katharina Linden, Stefan Kreyer, Gregory Beilman, Vittorio Scaravilli, Daniel Wendorff, Corina Necsoiu, Andriy I. Batchinsky, Leopoldo C. Cancio, Kevin K. Chung, Elizabeth R. Lusczek

**Affiliations:** 1 Department of Surgery, University of Minnesota, Minneapolis, MN 55455, USA; 2 US Army Institute of Surgical Research, Fort Sam Houston, TX 78234, USA; 3 Department for Pediatric Cardiology, University Hospital Bonn, 53113 Bonn, Germany; 4 Clinic for Anesthesiology and Intensive Care Medicine, University Hospital Bonn, 53127 Bonn, Germany; 5 Department of Anesthesia Critical Care and Emergency, Fondazione IRCCS Ca’ Granda Ospedale Maggiore Policlinico, 20122 Milan, Italy; 6 Department of Medicine, Uniformed Services University of the Health Sciences, Bethesda, MD 20814, USA

**Keywords:** metabolomics, burn, inhalation injury, 2-hydroxybutyrate, biomarker, NMR, Metabolism

## Abstract

Burn injury initiates a hypermetabolic response leading to muscle catabolism and organ dysfunction but has not been well-characterized by high-throughput metabolomics. We examined changes in metabolism over the first 72 h post-burn using proton nuclear magnetic resonance (^1^H-NMR) spectroscopy and serum from a porcine model of severe burn injury. We sought to quantify the changes in metabolism that occur over time in response to severe burn and smoke inhalation in this preliminary study. Fifteen pigs received 40% total body surface area (TBSA) burns with additional pine bark smoke inhalation. Arterial blood was drawn at baseline (pre-burn) and every 24 h until 72 h post-injury or death. The aqueous portion of each serum sample was analyzed using ^1^H-NMR spectroscopy and metabolite concentrations were used for principal component analysis (PCA). Thirty-eight metabolites were quantified in 39 samples. Of these, 31 showed significant concentration changes over time (*p* < 0.05). PCA revealed clustering of samples by time point on a 2D scores plot. The first 48 h post-burn were characterized by high concentrations of histamine, alanine, phenylalanine, and tyrosine. Later timepoints were characterized by rising concentrations of 2-hydroxybutyrate, 3-hydroxybutyrate, acetoacetate, and isovalerate. No significant differences in metabolism related to mortality were observed. Our work highlights the accumulation of organic acids resulting from fatty acid catabolism and oxidative stress. Further studies will be required to relate accumulation of the four organic carboxylates identified in this analysis to outcomes from burn injury.

## 1. Introduction

The metabolic response to major injury was described in 1942 by D.P. Cuthbertson as consisting of an “ebb” and a “flow” phase [1]. The ebb phase is marked by hypovolemic shock, hypometabolism, and a decrease in oxygen delivery and consumption. The flow phase dominates long-term burn recovery, and lean body mass loss due to increased metabolic cycling during this time is associated with organ dysfunction in severely burned patients. More recently, Xiao et al. published a seminal paper documenting a massive transcriptomic shift in human immune cells within the first 12 h of injury [2]. This shift from specific to innate immune gene expression was similar across mechanisms of injury, and it was particularly pronounced in burns. Circulating catecholamine levels have been correlated with a marked hypermetabolic response to burn injury [3], suggesting inflammation, catecholamine effects, and hypermetabolism are interrelated in burn recovery. 

The American Burn Association previously identified inhalation injury as a major contributor to morbidity and mortality from burn injury. In 2006, a working paper identified four key research priorities to improve diagnosis and grading of inhalation injury, to improve treatment and long-term outcomes associated with inhalation injury, and to improve understanding of mechanisms of injury and translational work through genomic, metabolomic, and proteomic investigations [4]. A decade later, a “State of the Science” review of these research priorities was published, summarizing the progress made in each topic area [5]. Despite a specific call for metabolomics investigations into inhalation injury, the review reported only genomics and proteomics studies. 

Thus, burn injury strongly impacts metabolism in a way that changes over time, and could benefit from high-throughput metabolomics studies, profiling changes in metabolism over time. Metabolomics is the study of the intermediates and end-products of metabolic activity in biological systems. Downstream of transcription and translation, the collected sum of metabolic substrates in a biological system reflects the breakdown, synthesis, and activity of protein produced to support cell function [6]. Metabolomics has been used in medical research to describe metabolic changes associated with trauma [7,8,9,10], cognitive impairment [11], and sepsis [12]. However, there are very few high-throughput metabolomics studies profiling changes in metabolism in the context of burn injury [13,14]. A second limitation of these studies is that biospecimens were only collected at a single timepoint. There is a clear need for a longitudinal metabolomics study of severe burn that includes an inhalation injury component.

Our goal in this study was to quantify changes in metabolism after burn and inhalation injury over time using proton nuclear magnetic resonance (^1^H-NMR) spectroscopy in a porcine model of severe burn injury and smoke inhalation. We hypothesized that serum metabolic profiles would change over time, and sought to create a coherent picture of how the metabolism changes over the first 72 h after burn. We further sought to identify potential biomarkers of injury to be explored in future research. 

## 2. Results

We performed a secondary analysis of serum samples from a study performed at the U.S. Army Institute of Surgical Research (USAISR) that utilized a porcine model of burn injury and smoke inhalation. Details of the study performed by USAISR, which sought to test the safety and efficacy of a particular hemoadsorption product for filtering cytokines from animals’ blood following severe burn injury and smoke inhalation, have been previously described [15]. 

Animal weight, survival, and indicators of burn injury severity (smoke breaths, peak carboxyhemoglobin) are shown in Table 1. No statistically significant differences were observed between sham and hemoadsorption groups. Thirty-nine serum samples were received from USAISR for analysis. (Supplementary Table S1). 

^1^H-NMR spectra (Supplementary Figure S1) were obtained for all 39 samples, and 38 metabolites were identified and quantified in each spectrum using Chenomx software. The resulting metabolic profiles were analyzed with principal component analysis (PCA) to assess latent variability among the samples by experimental group, survival, and experimental timepoint. PCA captured 51.6% of the variability in the data set in the first 2 principal components. Principal component 1 (PC1) accounted for 30.8% of variability, while principal component 2 (PC2) accounted for 20.8% of variability. 

PCA scores showed no clear clustering or separation of serum samples by treatment group or survival (Supplementary Figures S2 and S3, respectively). Only the metabolite creatinine differed significantly between sham and hemoadsorption animals (higher with hemoadsorption, *p* = 0.025). Only the metabolites 2-hydroxybutyrate, isoleucine, creatinine, lactate, and pyruvate differed between animals that survived the experiment and those that died before 72 h (0.003 < *p* < 0.05). Isoleucine and 2-hydroxybutyrate levels were higher in animals that lived, while creatinine, lactate, and pyruvate levels were higher in those that died. None of these differences remained statistically significant after Bonferroni correction. 

**Figure 1:**
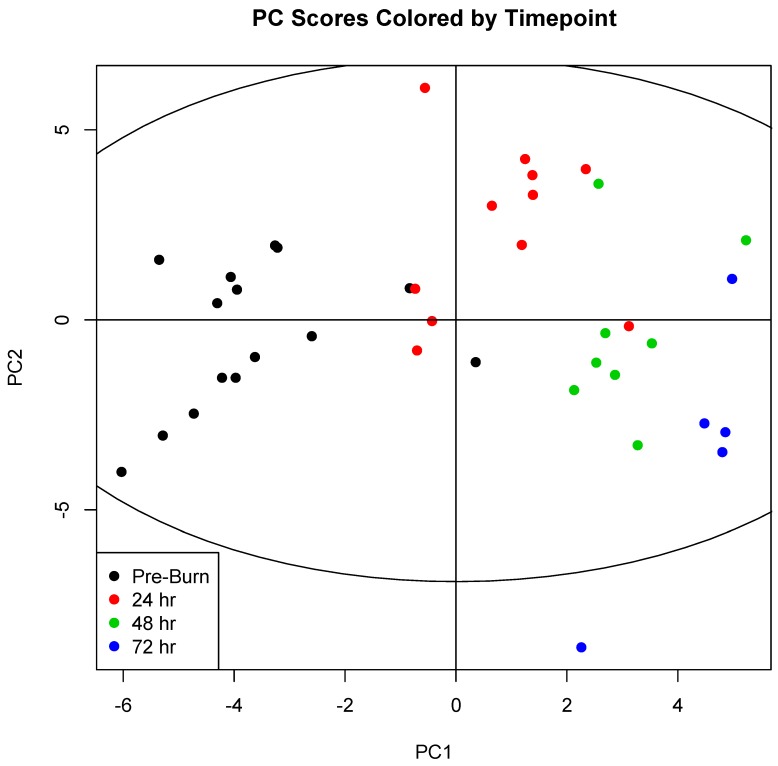
Principal component scores for components 1 (PC1) and 2 (PC2) were plotted for each sample. Samples were then colored by experimental timepoint to illustrate sample clustering. Comparison of this coloring scheme of the PCA scores with coloration by experimental group (Supplementary Figure S2) and survival (Supplementary Figure S3) illustrates a clear underlying variability explainable by changes in metabolism over time, and not by changes that accompany treatment with hemoadsoption or mortality. 0 h (*n* = 15); 24 h (*n* = 11); 48 h (*n* = 8); 72 h (*n* = 5).

On the other hand, PCA scores did show marked clustering of serum samples according to the experimental timepoint (Figure 1). Significant concentration changes (*p* < 0.05) were observed over time in 31 of 38 metabolites during the 72-h experimental period. Eighteen metabolites remained significant following Bonferroni correction (*p* < 0.0013; see Supplementary Table S2). 

**Figure 2:**
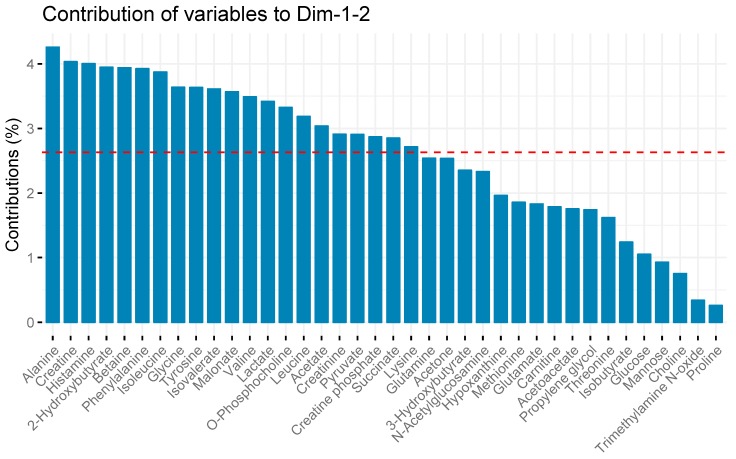
Metabolite contributions to serum sample variation in principal component analysis. The red line depicts the average amount of variability each metabolite contributes to overall variability captured in the PCA model. Metabolites with bars taller than the red line contributed more than the average, while metabolites with bars shorter than the red line contributed less. Metabolite contributions were calculated using the factoexta package, and are proportional to the sum of square of the loadings weighted by the eigenvalues.

Twenty-one metabolites contributed more than the average (2.63%) to the variability captured in the two-component model as shown in Figure 2. 

Because only one metabolite differed in concentration between the sham and treatment groups while the majority of them changed over time, and because PCA showed clear clustering according to the sample timepoint but not experimental group, we pooled the groups for subsequent data analysis and interpretation. Means and standard errors of the means for metabolite concentrations at each time point are reported in Table 2. 

**Figure 3:**
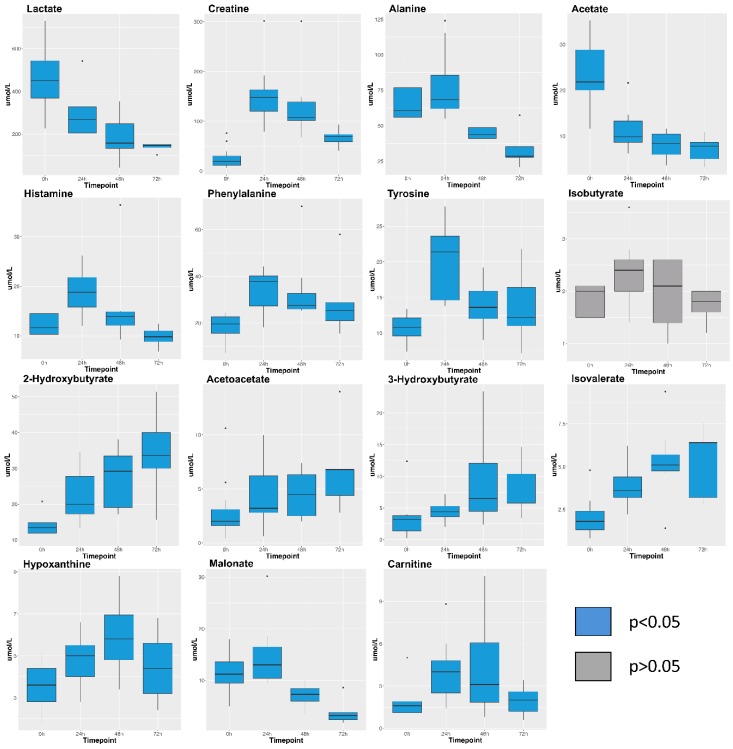
Box plots visualizing concentrations (micromolar) for selected metabolites. Plots show the median and interquartile range of profiled concentrations at each time point. Blue box plots indicate that metabolite concentration changed significantly during the experiment (*p* < 0.05). Grey box plots indicate the concentration of the metabolite did not change significantly (*p* > 0.05). Samples analysed per timepoint are: 0 h (*n* = 15); 24 h (*n* = 11); 48 h (*n* = 8); 72 h (*n* = 5). Kruskal-Wallis *p*-values for all metabolites can be found in Supplementary Table S2.

**Figure 4:**
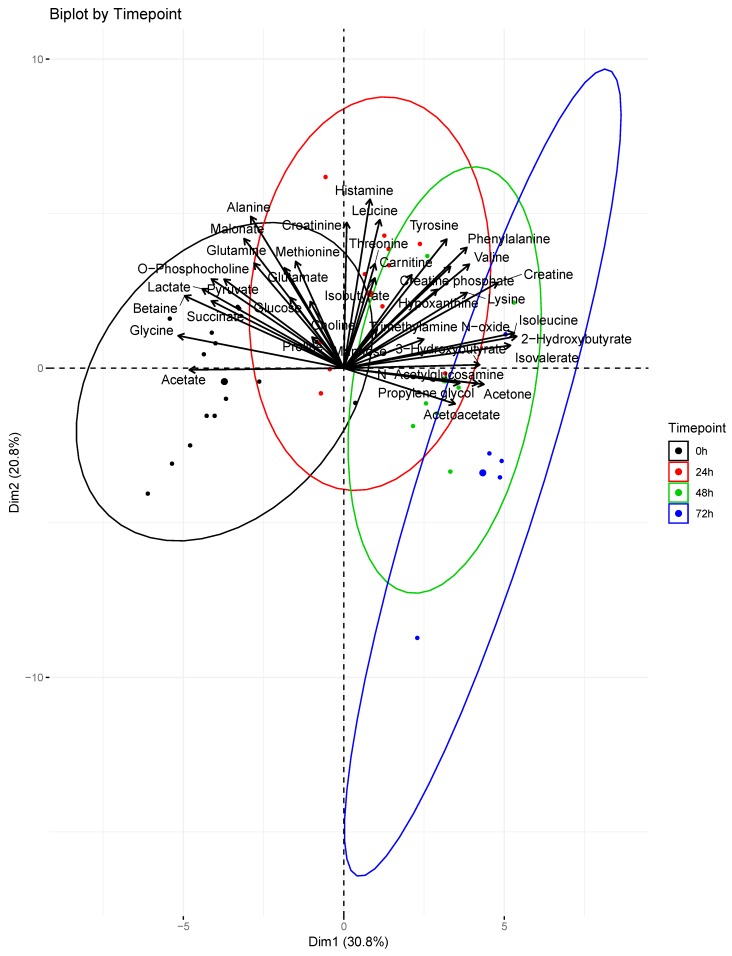
Principal component analysis biplot. Contributions of profiled metabolites were superimposed over scores for each serum sample. The direction and magnitude of the loadings vectors visually demonstrate how each metabolite contributes to sample clustering by timepoint. The larger dots represent the centroids of the ellipses calculated for each timepoint. At 0 h (*n* = 15); 24 h (*n* = 11); 48 h (*n* = 8); 72 h (*n* = 5).

Boxplots of several key metabolite concentrations with time are shown in Figure 3. A biplot (Figure 4) illustrates how individual metabolites contribute to the clustering of samples by timepoint. Early timepoints were characterized by high concentrations of histamine, alanine, phenylalanine, and tyrosine. Late timepoints were characterized by the accumulation of 2-hydroxybutyrate, 3-hydroxybutyrate, acetoacetate, and isovalerate. 

## 3. Discussion

This secondary metabolomic evaluation of serum from a porcine model of burn injury and smoke inhalation found no significant differences in serum metabolic profiles in either treatment group (sham vs. hemoadsorption) or survival. As previously reported in Linden et al., though hemoadsorption removed cytokines from the blood, it did not significantly change systemic cytokine levels, nor did it have any effect on the hemodynamic, pulmonary, or hematologic variables measured [15]. Based upon these results and the results presented here, we are justified in pooling the groups when interpreting our findings. The clear effect of the experimental timepoint on metabolite concentrations and principal component analysis frames our interpretation of the data. 

Principal component analysis showed a striking pattern in serum sample clustering according to experimental timepoint, with a progressive shift from pre-burn samples through 24, 48, and 72 h after injury. This was reflected in an abundance of metabolites that showed statistically significant variation over time. Over 80% of profiled metabolites changed over time at the level of *p* < 0.05. After Bonferroni correction, 47% remained statistically significant. This study reflects the metabolic progression that accompanies resuscitation from burn injury, a concept first explored by Cuthbertson in 1942 [1].

Histamine, creatine, and several amino acids explain most of the variation in the first 48 h of our model. Patterns in the concentration changes of these metabolites reflect what is currently known about early changes in metabolism as a result of injury. Histamine concentrations peaked at 24 h and were then cleared from the circulation (Figure 3), potentially indicating an initial increase in vascular permeability followed by a decrease. Histamine is released by mast cells in damaged tissues, and it contributes to burn shock by disrupting tight junctions in vascular epithelia [16]. It has been quantified in both animal models of burn injury [17] and in the serum of human burn patients [18]. Creatine concentrations also peaked at 24 h caused by catabolism after burn injury [19]. Alanine concentrations peaked at 24 h. Alanine is a major carrier of amino acid carbons from muscle to the liver for gluconeogenesis [20]. Tissue damage from deep burns and inflammation release alanine from muscle cells immediately following injury [3]. Decreasing concentrations thereafter were likely due to increased amino acid cycling for gluconeogenesis [21]. Phenylalanine and tyrosine concentrations also peaked at 24 h. Phenylalanine is a precursor for tyrosine synthesis, and tyrosine is necessary for production of catecholamines including epinephrine and dopamine. Catecholamines are required for maintaining hypermetabolism in burn injury, and increased tyrosine cycling is known to occur in response to burn trauma [21,22,23].

**Figure 5:**
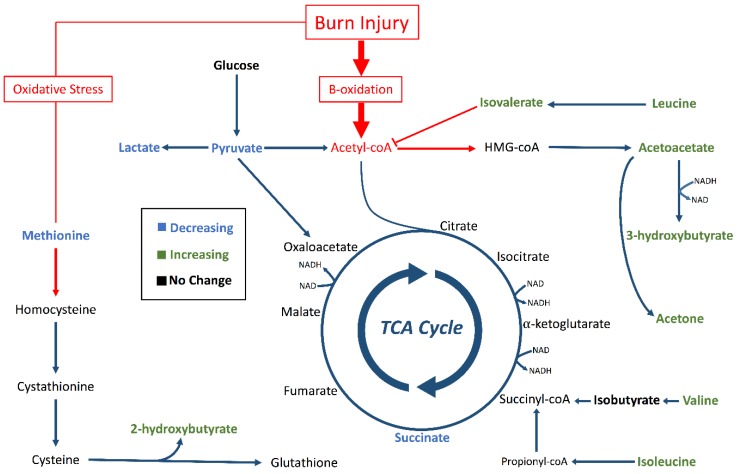
Metabolic pathways altered by burn injury. Pathways for ketogenesis, the TCA cycle, and transsulfuration for glutathione synthesis were outlined with focus on metabolites identified by in our analysis (bold). Green metabolites had significantly increasing concentration trends (*p* < 0.05). Blue metabolites had significantly decreasing concentration trends (*p* < 0.05). Black metabolites did not change concentration significantly (*p* < 0.05), and non-bolded metabolites were not identified in our analysis. The red arrows and boxes represent possible aberrations due to burn injury that could have resulted in accumulation of 2-hydroxybutyrate, isovalerate, acetoacetate, and 3-hydroxybutyrate.

Four organic carboxylates (2-hydroxybutyrate, isovalerate, acetoacetate, 3-hydroxybutyrate) increased in concentration steadily from 0 to 72 h (Figure 3). PCA associated these metabolites with later timepoints (48–72 h, Figure 4). We propose that oxidative stress and fatty acid catabolism increase over time, leading to anion accumulation. We constructed a metabolic scheme (Figure 5) to explain this interpretation of our data.

2-hydroxybutyrate was the fourth most significant contributor to sample clustering in our PCA model, contributing strongly to the 72-hour cluster of samples (Figure 4). This may be due to oxidative stress resulting from reperfusion following burn shock [24]. 2-hydroxybutyrate is produced during cysteine synthesis, which is needed to sustain increased glutathione cycling during times of high oxidative stress [25]. Increasing hypoxanthine concentrations through 48 h provide additional evidence of oxidative stress following resuscitation. Hypoxanthine is a substrate of xanthine oxidase, which produces hydrogen peroxide as a byproduct in purine breakdown [26]. Xanthine oxidase activity is known to increase following burn injury, and increased serum hypoxanthine levels are markers of increased purine catabolism [27]. Taken together, increasing concentrations of hypoxanthine and 2-hydroxybutyrate suggest post-resuscitation oxidative stress necessitates increased glutathione cycling in burn injury, leading to 2-hydroxybutyrate accumulation.

Isovalerate accumulation also contributed significantly to the clustering of samples at later timepoints in our PCA model. Isovalerate is an intermediate of leucine metabolism that accumulates in the blood in conditions of incomplete leucine breakdown [28]. However, isobutyrate, the analog to isovalerate in valine catabolism, did not show significant concentration changes during the study period (Figure 3). We interpret this to mean that leucine catabolism was disturbed following burn injury, but valine catabolism proceeded unchanged. Importantly, isovalerate enters the TCA cycle as acetyl-CoA and isobutyrate enters the cycle as succinyl-CoA [29]. We propose that, under conditions of high acetyl-CoA production such as those following burn injury, conversion of isovalerate to acetyl-CoA is inhibited while isobutyrate conversion to succinyl-CoA continues unaffected (Figure 5). This results in isovalerate accumulation in resuscitated burn victims.

Acetoacetate and 3-hydroxybutyrate both increased significantly during later timepoints. These ketone bodies are used by vital organs during times of metabolic stress or increased fatty acid catabolism. In diabetes, accumulation of ketone bodies occurs due to the effects of insulin resistance and increased dependence on fats as fuels [30]. In starvation, ketosis occurs because carbohydrate stores are depleted and fats are the major remaining fuel source to support organ function [31]. Burn injury combines these two etiologies, where the early “fight-or-flight” response depletes glycogen stores and causes insulin resistance in skeletal muscle cells. Both starvation and burn injury impact ketone body levels, with burn injury blunting the initial elevation of ketones [32]. 

This model suggests increased fatty acid breakdown in response to burn injury creates an abundance of acetyl-CoA (Figure 5). Excess acetyl-CoA is converted into ketone bodies, and high acetyl-CoA concentrations inhibit leucine breakdown. Concurrently, oxidative stress following resuscitation from burn shock leads to increased glutathione cycling, which creates 2-hyrdoxybutyrate as a byproduct. In this way, 2-hydroxybutyric acid, isovaleric acid, acetoacetic acid, 3-hydroxybutyric acid all accumulate following resuscitation in burn victims. 

Two other metabolites, malonate and carnitine, are regulators of fatty acid metabolism that showed strong covariance in our PCA model. Both carnitine and malonate concentrations peaked at 24 h (Figure 3). Malonate is the carboxylate counterpart to malonic acid and could be an early contributor to acidosis that resolves following resuscitation. However, there is little published about mammalian production of malonate other than it accumulates with deficiencies of malonyl-CoA decarboxylase, an enzyme that converts malonyl-CoA to actetyl-CoA to promote increased β-oxidation [33]. Carnitine, which promotes β-oxidation as a shuttle for fatty acids into mitochondria, followed a similar pattern to malonate. 

There is a rich body of literature describing the effect of burn injury on liver and muscle [34,35,36,37,38]. For instance, liver size markedly increases immediately post-burn, and remains enlarged through at least three weeks post-burn [35]. Burn injury profoundly changes metabolism in these tissues, and these well-described changes likely influence the serum metabolome. Herndon et al. describe increased oxygen consumption in these tissues, leading to increased energy expenditures in burn patients. Glucose, protein, and fatty acid oxidation were all markedly increased after burn [34]. Increased protein catabolism has been shown to occur particularly in the muscle, persisting through at least three weeks post-burn [35]. Fatty acid oxidation increased the most, at 132% above healthy individuals. This fits well with our description of increased fatty acid catabolism. 

We faced several limitations in this study. This was a secondary analysis of serum samples obtained from a study meant to evaluate the feasibility, safety, and efficacy of cytokine and myoglobin removal. Relatively few animals were used in the study and it is exploratory in nature. All results and conclusions should be interpreted in light of this. The results of this study will be used to inform power calculations for future studies designed to evaluate outcomes such as mortality, as the current study was not powered to do so. In the context of inhalation injury, a limitation of our study is that we cannot evaluate the effects of inhalation injury alone on the metabolome. We focused on aqueous metabolites in the serum, but a study of lipid metabolites would be important to explore our findings and conclusions related to fatty acid catabolism. Likewise, we did not quantify acetyl-CoA and thus we are unable to verify our assertions concerning this molecule. The metabolites histamine and histidine have very similar spectral signatures and our identification of histamine should be validated in future work. A potential minor limitation is that there is a small difference in animal weight between the two groups. Finally, the data collected at later timepoints reflect only animals that survived to the end of the experiment, introducing bias into the interpretation of our data. Future studies should include fasted controls to account for the effects of animals’ fasted state on increases in ketones. Non-injured controls should also be part of future studies to ensure that observed changes to metabolism are due to injury. Future work focused on relating increases in the four organic carboxylates to resuscitation endpoints or patient outcomes is important to explore their utility as biomarkers. 

## 4. Materials and Methods 

All animal protocols were approved by the USAISR Animal Care and Use Committee, and were compliant with the Animal Welfare Act and The Guide for the Care and Use of Laboratory Animals [39]. Animals were not used for any other experiments and were otherwise healthy at the time of this study. Animals were euthanized at the end of the study with 10 mL of a euthanasia solution (Fatal-Plus, Med-Vet International, Mettawa, Illinois) in accordance with the American Veterinary Medical Association Guidelines on Euthanasia from June, 2007 [40].

Fasted female, non-pregnant, adult Yorkshire pigs (*n* = 15) were used in this study. Females were used to facilitate urinary catheterization. All animals were anesthetized initially with tiletamine/zolazepam (6 mg/kg) followed by inhaled isoflurane (1–3 Vol %). This was followed by total intravenous anesthesia for the remainder of the experiment with fentanyl (5–15 µg/kg/h), midazolam (0.1–0.5 µg/kg/h), ketamine (10–30 mg/kg/h) and propofol (0.5–3 mg/kg/h). Catheters were placed in the left carotid artery and in the pulmonary artery via the left jugular vein. A dialysis catheter was placed in the right jugular vein for extracorporeal blood treatment. Tracheostomy was performed to administer smoke inhalation injury. Pine bark was burned in a custom chamber and cooled prior to administration via tracheostomy. Tidal volume per smoke breath was set at 30mL/kg body weight with a desired endpoint of 80–90% arterial carboxyhemoglobin as determined by blood gas analysis. A Bunsen burner was used to apply full-thickness burns to each flank, 20% total body surface area (TBSA) on each side. Animals were transferred to the animal ICU, where they were resuscitated with lactated Ringer’s guided by computerized decision support software [41]. This was transitioned to a maintenance infusion of 0.45% sodium chloride and 5% glucose to a target urine output of 0.5–1 mL/kg/h following stabilization or at 48 h post-injury. Moribund animals tended to have acute hypoxia or unresolvable renal failure as determined by a clinician and were euthanized with Fatal Plus. Two animals died due to arrhythmias before Fatal Plus could be administered.

Animals were randomized to extracorporeal blood treatment groups that used either a CytoSorb hemoadsorption column (*n* = 9) (CytoSorbents Corporation, Monmouth Junction, New Jersey) or a sham extracorporeal circuit (*n* = 6) beginning at noon. Animals underwent 6-hour extracorporeal treatment sessions starting immediately after burn treatment and at 24-hour intervals thereafter. Blood was drawn before burn (0 h) and prior to daily extracorporeal treatment through 72 h or until death. Serum was extracted and stored at −80 °C. 

Serum was transported on dry ice and stored at −80 °C until analysis. Samples were thawed and filtered using 3 kDa Centrifee*®* centrifugal filters (Merck Millipore Ltd, Darmstadt, Germany) at 4 °C and 6000 rpm for 2–3 h; 250 μL filtrate was combined with 250 μL sodium phosphate buffer prepared with D_2_O. Then, 50 μL of 3-(trimethylsilyl)propionic acid (TSP, Sigma-Aldrich, St. Louis, MO, USA) was added to a concentration of 0.5 mM as an internal standard. Final pH was recorded and the solution was transferred to 5 mm NMR tubes for immediate analysis.

1H-NMR spectra were collected using a Bruker Avance spectrometer with autosampler and 5 mm triple resonance 1H, 13C, and 15N TXI CryoProbe with Z-gradient running TopSpin v. 2.16 (Bruker BioSpin, Fremont, CA, USA) at 700.13 MHz. A 1D Carr-Purcell-Meiboom-Gill (CPMG) pulse sequence was used with a relaxation time defined by the 90° pulse width calibrated for each sample. Relaxation delay was 2 s, acquisition time was 3 s, spectral width was 10 KHz, total data points collected were 63,000, and number of transients collected were 128. All spectra were collected at 298 K.

All spectra were analyzed using Chenomx software (Edmonton, AB, Canada) [42]. Manual phasing, baseline correction, and the Chenomx Reference Deconvolution algorithm were applied before profiling was performed. Identified metabolites were fit to each spectrum by the same human operator, resulting in sample profiles consisting of each metabolite and its concentration in millimoles per liter (mM).

Statistical analysis was performed using R software [43]. Principal component analysis (PCA) was used for multivariate statistical analysis. Metabolite concentrations were log-transformed and scaled to unit variance prior to use in PCA. The FactoMineR package [44] was used for PCA calculation and the factoextra package [45] was used for ggplot2-based visualization of PCA outputs. The first two principal component scores (PC1/PC2) for each sample were projected onto an X-Y plot for visual interpretation. Ranked contributions of individual metabolites to variability captured in the two-component model were calculated using contributions to variability in the first two principal components weighted by the eigenvalues for those principal components.

Statistical significance of individual metabolites was evaluated using Kruskal-Wallis or Wilcoxon Rank Sum testing. Bonferroni multiple test correction was used to designate a subgroup of metabolites with significant trends after adjustment for 38 tests (*p* < 0.0013). 

## 5. Conclusions

We found no significant differences in metabolism related to either hemoadsorption treatment to remove cytokines following burn injury or mortality in this preliminary study. However, we did find a clear, significant shift in metabolism over the first 72 h following burn injury, with a majority of metabolites changing in concentration over time. The initial response to injury (24–48 h) is dominated by changes in histamine levels and amino acids which have already been documented in the literature. We propose that the later changes in metabolism (48–72 h) are driven by a steady increase in fatty acid catabolism and oxidative stress, leading to the accumulation of four organic carboxylates (2-hydroxybutyrate, isovalerate, acetoacetate, 3-hydroxybutyrate) that increased in concentration from pre-burn to 72 h post-burn. These metabolites can be explored in future work as biomarkers of resuscitation or recovery from burn injury, particularly if they can be related to outcomes. 

## Figures and Tables

**Table 1:** Subject data are displayed as mean ± standard error of the mean.

Treatment Group	Weight (kg)	Smoke Breaths	Peak COHb (%)	Survival (h after Injury)
Hemoadsorbtion (*n* = 9)	41.3 ± 1.6	22 ± 0.4	85.3 ± 2.0	31.3 ± 6.5
Sham (*n* = 6)	38.8 ± 1.1	21 ± 0.6	86.0 ± 3.8	57.3 ± 3.4
*p*-value	0.26	0.14	0.86	0.054

*p*-values were based on t-testing for weight, smoke breaths, and peak COHb using log rank testing for survival. COHb, carboxyhemoglobin. This information was previously published by Katharina Linden, Vittorio Scaravilli, Stefan Kreyer, et al., “Evaluation of the Cytosorb™ Hemoadsorptive Column in a PIG Model of Severe Smoke and Burn Injury”, Shock Injury, Inflammation and Sepsis, Volume 44 Issue 5, and is reproduced with permission. The Creative Commons license does not apply to this content. Use of the material in any format is prohibited without written permission from the publisher, Wolters Kluwer Health, Inc. Please contact permissions@lww.com for further information.

**Table 2:** Mean concentrations (μM) and standard errors of the means (μM) for individual metabolites at each time point.

Metabolite	0 h Mean (*n* = 15)	0 h SE	24 h Mean (*n* = 11)	24 h SE	48 h Mean (*n* = 8)	48 h SE	72 h Mean (*n* = 5)	72 h SE
**2-Hydroxybutyrate**	14.0	0.7	22.3	2.1	27.3	2.9	34.1	5.9
***3-Hydroxybutyrate***	3.2	0.7	4.5	0.4	9.1	2.5	8.0	2.0
**Acetate**	23.3	1.8	11.2	1.3	8.1	1.0	7.1	1.3
***Acetoacetate***	2.8	0.7	4.5	0.8	4.6	0.8	7.0	1.9
**Acetone**	2.2	0.3	8.7	5.8	17.1	2.4	24.0	8.2
**Alanine**	65.9	4.3	77.5	7.2	43.8	1.3	33.8	6.3
**Betaine**	44.7	3.5	31.3	4.9	10.5	1.3	4.1	1.1
***Carnitine***	1.7	0.3	4.0	0.6	4.3	1.3	2.0	0.5
**Choline**	2.3	0.3	1.8	0.3	2.3	0.5	2.0	0.3
**Creatine**	25.0	5.1	151.9	18.0	133.0	25.6	67.2	8.7
***Creatine phosphate***	5.3	0.6	9.0	1.0	10.8	3.8	7.6	1.5
***Creatinine***	14.9	0.9	19.8	2.2	20.0	5.5	10.4	1.4
**Glucose**	697.4	45.3	669.2	43.8	653.4	63.9	514.7	55.9
***Glutamate***	23.9	2.0	22.4	2.2	20.1	3.2	15.3	1.3
**Glutamine**	38.0	4.0	33.1	3.4	26.6	5.1	16.5	1.5
**Glycine**	140.7	10.2	78.2	9.0	43.1	5.3	36.5	8.4
**Histamine**	12.3	0.7	19.0	1.3	15.8	3.0	9.8	1.0
***Hypoxanthine***	3.6	0.3	4.8	0.4	6.0	0.6	4.5	0.8
**Isobutyrate**	1.9	0.1	2.4	0.2	2.0	0.2	1.7	0.1
**Isoleucine**	14.2	0.7	23.8	2.4	27.5	3.0	35.5	6.2
**Isovalerate**	2.0	0.3	3.9	0.4	5.3	0.8	5.3	1.0
**Lactate**	458.1	35.6	285.4	31.7	187.3	35.2	139.8	9.7
***Leucine***	21.4	1.5	31.4	3.2	21.2	2.5	20.2	5.8
***Lysine***	10.3	1.1	17.8	1.8	20.2	2.5	17.0	4.9
**Malonate**	11.4	0.9	14.6	1.8	7.0	0.8	4.0	1.2
**Mannose**	5.5	0.6	9.8	0.9	9.2	0.8	10.4	1.2
***Methionine***	6.1	1.0	7.1	0.6	3.5	0.5	3.9	1.3
***N-Acetylglucosamine***	2.2	0.6	4.4	0.8	7.0	1.4	8.8	2.8
**O-Phosphocholine**	1.9	0.2	1.4	0.2	1.0	0.1	0.6	0.1
**Phenylalanine**	18.5	1.3	34.1	2.6	34.0	5.4	29.8	7.4
**Proline**	16.3	2.9	19.6	3.2	11.2	1.8	10.0	2.3
***Propylene glycol***	6.7	1.8	9.6	3.0	18.7	4.0	27.0	9.5
**Pyruvate**	21.3	2.2	16.9	2.1	10.0	0.8	8.2	0.9
**Succinate**	2.8	0.3	1.9	0.2	1.4	0.1	1.2	0.1
**Threonine**	17.2	1.4	21.5	2.8	19.6	1.8	14.7	2.6
**Trimethylamine-N-oxide**	4.7	1.4	5.4	1.4	5.5	1.4	5.3	1.8
**Tyrosine**	10.7	0.5	20.3	1.6	13.9	1.2	13.7	2.5
***Valine***	35.6	1.6	59.1	4.9	49.3	5.3	57.5	12.6

Concentrations are not adjusted for dilution with buffer. Kruskal-Wallis testing was used to test whether concentrations changed significantly over time. Bold text indicates statistical significance with Bonferroni correction (*p* < 0.0013). Italicized text indicates statistical significance at *p* < 0.05. Plain text indicates no statistical significance.

## Supplementary Materials

The following are available online at https://www.mdpi.com/2218-1989/9/7/142/s1, Figure S1: Sample NMR spectrum, Figure S2: Principal component scores for components 1 (PC1) and 2 (PC2) colored by experimental group (black=sham, red=hemoadsorption), Figure S3: Principal component scores for components 1 (PC1) and 2 (PC2) colored by survival (black=lived, red=died); Table S1: Sample Collection by Group and Timepoint., Table S2: Metabolite *p*-values. Availability of data and materials: Raw metabolite concentrations (mM) are available through the Data Repository for the University of Minnesota. Link: https://conservancy.umn.edu/handle/11299/199794.
