# Supplementary material for: 1H-NMR Metabolomics Identifies Significant Changes in Metabolism over Time in a Porcine Model of Severe Burn and Smoke Inhalation
Source: Metabolites. 2019 Jul 12;9(7):142. doi: 10.3390/metabo9070142 (PMC6680385; doi:10.3390/metabo9070142)
Supplement: Supplementary file 1 [file metabolites-09-00142-s001.pdf]

Supplementary Materials

Figure S1: Example NMR spectrum of an animal's sample collected at 24 h post-burn. Peaks are marked with identifications made with Chenomx software.

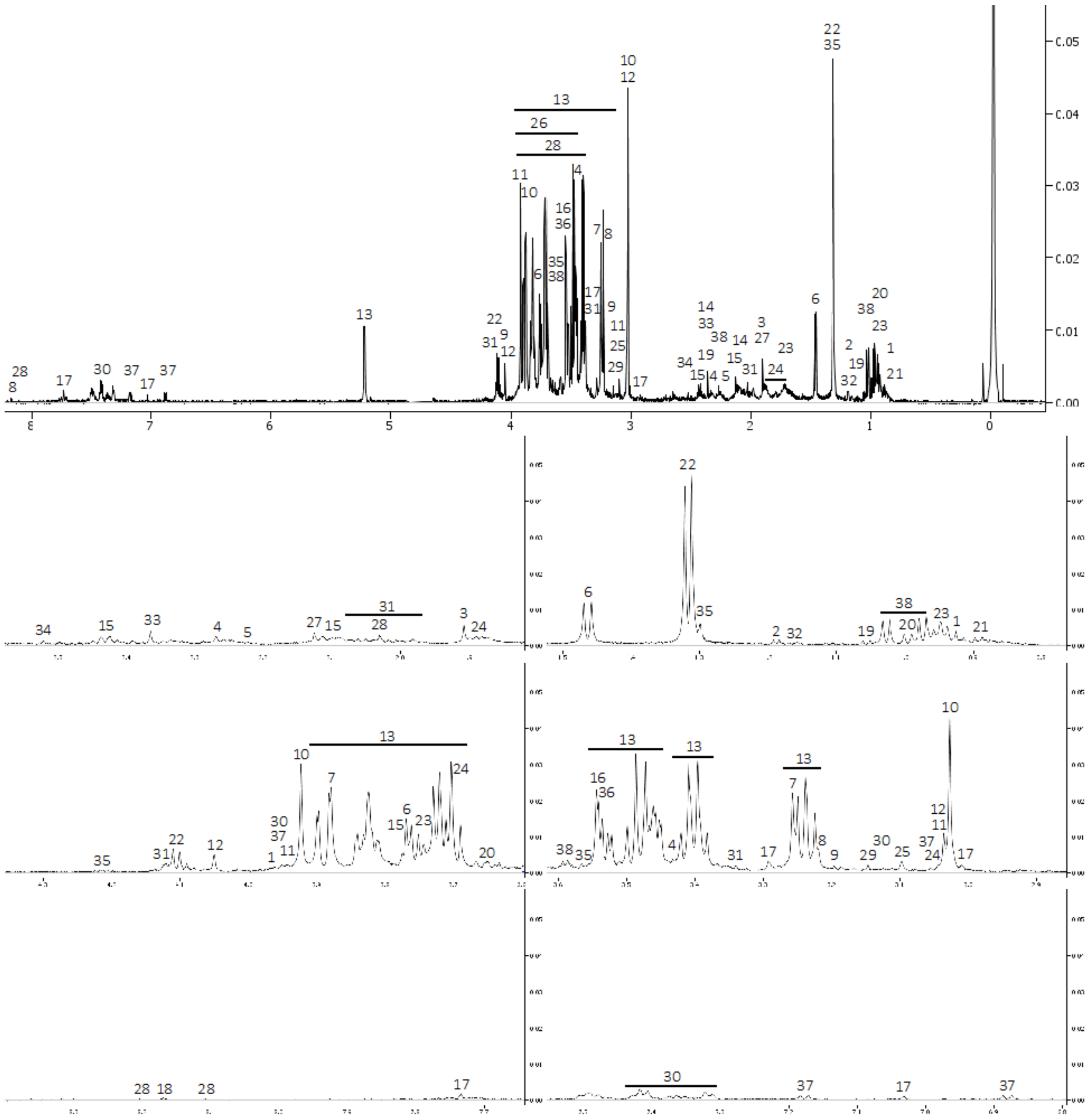

Figure S1. Known peaks from the Chenomx database were matched to ^{1}H-NMR spectra to obtain concentration values relative to the internal standard TSP. Concentrations shown here were not adjusted for dilution or loss to filtration. Peaks are marked by metabolite number listed alphabetically as follows: 1) 2-hydroxybutyrate, 2) 3-hydroxybutyrate, 3) acetate, 4) acetoacetate, 5) acetone, 6) alanine, 7) betaine, 8) carnitine, 9) choline, 10) creatine, 11) creatine phosphate, 12) creatinine, 13) glucose, 14) glutamate, 15) glutamine, 16) glycine, 17) histamine, 18) hypoxanthine, 19) isobutyrate, 20) isoleucine, 21) isovalerate, 22) lactate, 23) leucine, 24) lysine, 25) malonate, 26) mannose, 27) methionine, 28) N-acetylglucosamine, 29) O-phosphocholine, 30) phenylalanine, 31) proline, 32) propylene glycol, 33) pyruvate, 34) succinate, 35) threonine, 36) trimethylamine-N-oxide, 37) tyrosine,

38) valine. Position on the x-axis is determined by chemical shift in parts per million (ppm). The spectrum depicted above was collected from an animal at 24-hours following burn injury.

Figure S2: Principal component scores for components 1 (PC1) and 2 (PC2) were plotted for each sample. Each dot corresponds to a serum sample. Samples were colored according to experimental group (black=sham (*n* = 18), red=hemoadsorption (*n* = 21)). A 95% confidence ellipse surrounds the scores. No clear pattern in experimental group was observed.

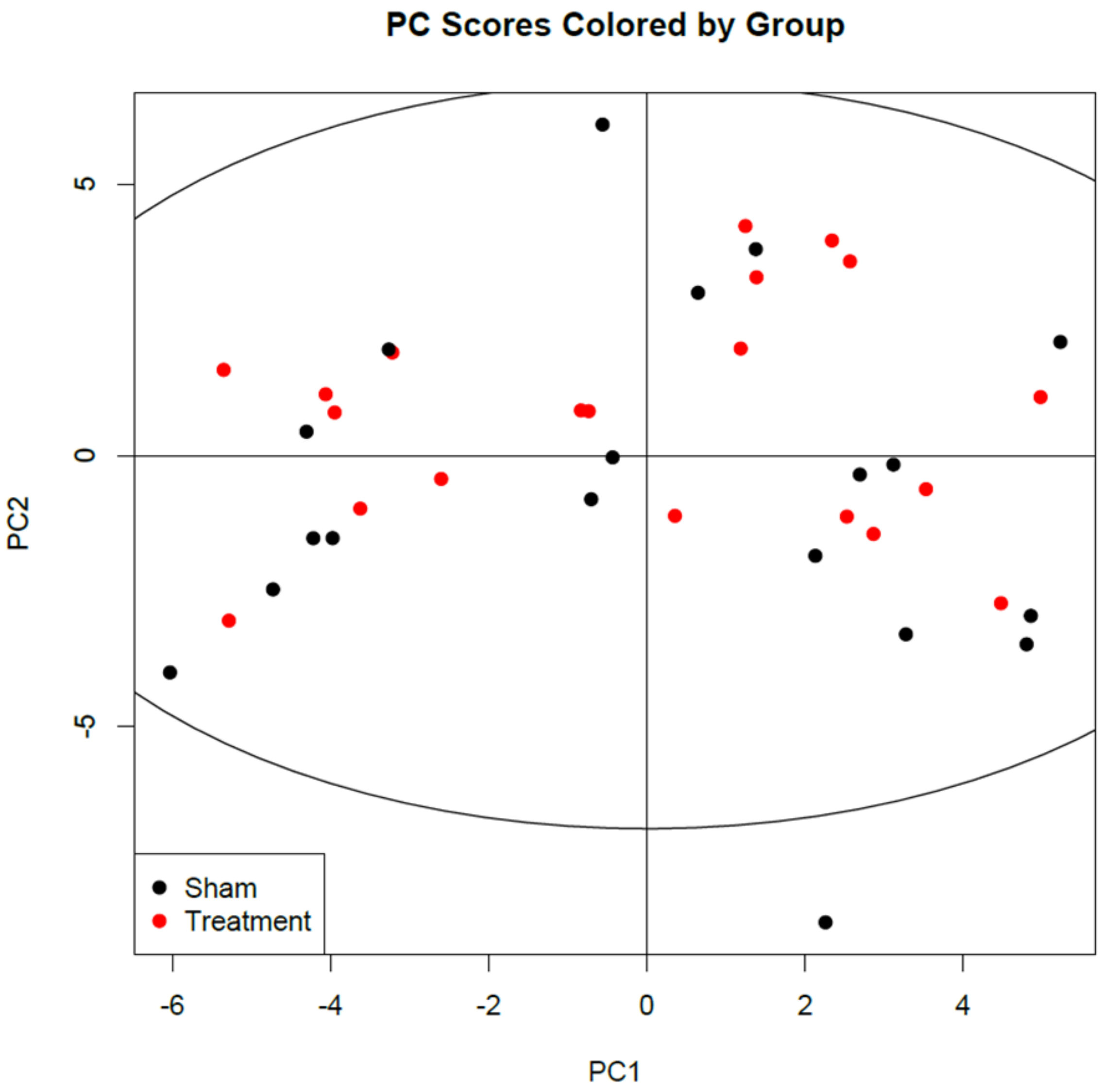

Figure S2. Principal component scores for components 1 (PC1) and 2 (PC2) colored by experimental group (black=sham, red=hemoadsorption).

Figure S3: Principal component scores for components 1 (PC1) and 2 (PC2) were plotted for each sample as in Supplemental Figure 1. Here, however, samples were colored according to survival (black=lived (*n* = 20), red=died (*n* = 19)). A 95% confidence ellipse surrounds the scores. No clear pattern in survival was observed.

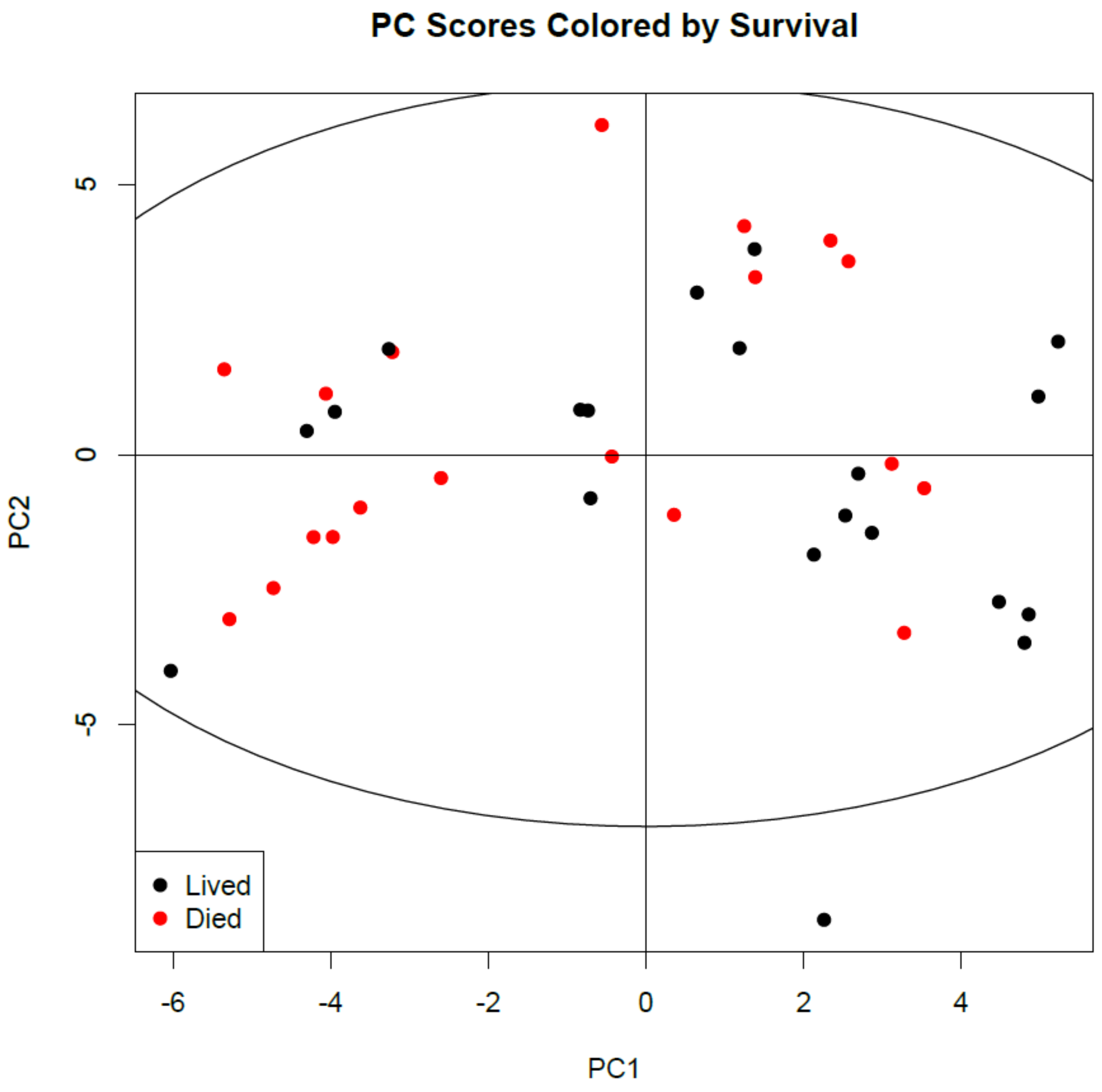

Figure S3. Principal component scores for components 1 (PC1) and 2 (PC2) colored by survival (black=lived, red=died).

Table S1. Sample Collection by Group and Timepoint. Serum samples were collected from surviving subjects at 0 h (pre-burn), 24 h, 48 h, and 72 h. Thirty-nine samples were collected in total.

Time Point	Hemoadsorption	Sham	Total Samples
0h	9	6	15
24h	6	5	11
48h	4	4	8
72h	2	3	5

Table S2. Metabolite p-values. The significance of individual metabolite concentration trends over time was calculated with Kruskal-Wallis tests. Significance was accepted when $p < 0.05$. Bold metabolites had concentration changes that remained significant following Bonferroni multiple test correction ($p < 0.0013$). Metabolites are ordered by contribution to variability as shown in Figure 2.

Metabolite	KW *p*-value	Metabolite	KW p-value
**Alanine**	**0.0001**	**Succinate**	**0.0005**
**Creatine**	**<0.0001**	Lysine	0.0034

**Histamine**	**0.0003**	Glutamine	0.0110
**2-Hydroxybutyrate**	**0.0001**	**Acetone**	**0.0011**
**Betaine**	**<0.0001**	3-Hydroxybutyrate	0.0037
**Phenylalanine**	**0.0001**	N-Acetylglucosamine	0.0052
**Isoleucine**	**0.0001**	Hypoxanthine	0.0157
**Glycine**	**<0.0001**	Methionine	0.0044
**Tyrosine**	**0.0001**	Glutamate	0.1199
**Isovalerate**	**0.0002**	Carnitine	0.0136
**Malonate**	**0.0001**	Acetoacetate	0.0306
Valine	0.0033	Propylene glycol	0.0033
**Lactate**	**<0.0001**	Threonine	0.3195
**O-Phosphocholine**	**0.0011**	Isobutyrate	0.0983
Leucine	0.0341	Glucose	0.1613
**Acetate**	**<0.0001**	**Mannose**	**0.0006**
Creatinine	0.0025	Choline	0.6691
**Pyruvate**	**0.0001**	Trimethylamine-N-oxide	0.7573
Creatine phosphate	0.0195	Proline	0.1769
